# Au_19_M (M=Cr, Mn, and Fe) as magnetic copies of the golden pyramid

**DOI:** 10.1038/s41598-017-16412-3

**Published:** 2017-11-22

**Authors:** Nguyen Minh Tam, Ngo Tuan Cuong, Hung Tan Pham, Nguyen Thanh Tung

**Affiliations:** 1 Computational Chemistry Research Group, Ton Duc Thang University, Ho Chi Minh City, Vietnam; 2 Faculty of Applied Sciences, Ton Duc Thang University, Ho Chi Minh City, Vietnam; 3 Faculty of Chemistry and Center for Computational Science, Hanoi National University of Education, Hanoi, Vietnam; 4 Department of Chemistry, KU Leuven, Celestijnenlaan 200F, B-3001 Leuven, Belgium; 5 Institute of Materials Science and Graduate University of Science and Technology, Vietnam Academy of Science and Technology, Hanoi, Vietnam

## Abstract

An investigation on structure, stability, and magnetic properties of singly doped Au_19_M (M=Cr, Mn, and Fe) clusters is carried out by means of density functional theory calculations. The studied clusters prefer forming magnetic versions of the unique tetrahedral Au_20_. Stable sextet Au_19_Cr is identified as the least reactive species and can be qualified as a magnetic superatom. Analysis on cluster electronic structures shows that the competition between localized and delocalized electronic states governs the stability and magnetic properties of Au_19_M clusters.

## Introduction

Last decades have witnessed an increasing interest in gold nano-clusters thanks to their precious catalytic, electronic, and optical properties^1–3^. In this respect, geometric and electronic structures of gold clusters with different sizes and charge states have been studied intensively through both theoretical simulations^4–8^ and experiments, for instance, infrared vibrational spectroscopy^9–11^, photoelectron spectroscopy^12–14^, photoionization spectroscopy^15,16^, photodissociation^17–19^, and ion mobility measurements^20,21^. The cluster form of gold containing low-coordinated edge atoms^22^ can adopt binding geometries and lead to a strong reactivity that differs from the bulk^23^. Enhanced stabilities were observed when the number of gold atoms corresponds to the following “magic” values: 2, 8, 18, 20, 34, 58, and 92, where the electronic supershells of clusters are supposed to be closed^24–29^. A rich structural diversity compared to other coinage metals has been reported for gold clusters due to their strong relativistic effects. It has been revealed that small Au_*n*_ clusters prefer planar structures till the cluster size of 8–13 atoms depending on charge states^20,30–34^. Larger species evolve via hollow cage structures at *n* = 14–18^9,35–37^, tube-like structure at *n* = 24–26^38,39^, fullerene type at Au_32_^40,41^, and a possibly chiral structure at *n* = 34^8^. Among the wide range of studied gold nanoclusters, the discovery of the tetrahedral Au_20_, as an outstanding landmark in cluster science, has attracted an exceptional attention than any others^9,12,42,43^. It was reported that Au_20_ possesses a perfect pyramidal structure (*T*_*d*_) with a considerably large highest occupied molecular orbital-lowest unoccupied molecular orbital (HOMO-LUMO) energy gap of 1.77 eV, suggesting its remarkably high stability and offering a potential platform for optical and catalytic nanostructured materials. The underlying physics behind its magic stability has been attributed to the formation of the 20-electron supershell closure in which each Au atom contributes one itinerant 6*s* electron^42^. Au_20_ can also be considered as either a group of ten 2-electron superatoms in superatom-network model or a superatomic molecule Au_16_ bonded with four vertex Au atoms^44^.

Interests in synthesizing novel elementary units for advanced nano-structured materials led to extensive search for stable cluster species with desired properties. A presence of impurity (i.e. alkali, coinage, and transition metal) atoms can provide a key to tune the properties of Au_20_ clusters with minor effects on its magic stability. In this regard, the structures, binding energies, ionization potentials, electron affinities, and energy gaps of Au_19_X (X = Li, Na, K, Rb, Cs, Cu, and Ag) were systematically examined^45^. Au_19_Li, Au_19_Cu, Au_19_Ag, and Au_19_Pt are found to retain the pyramidal geometry of Au_20_^46–51^. Increasing the Ag concentration induces the energy gap and optical transition of Au_19−*n*_Ag_*n*_^48,49^. Au_19_Cu and Au_19_Pd clusters are potential for adsorption of trivalent arsenic^50^. Substitutional doping of Pt in Au_20_ leads to significant enhancement of the cluster binding energy and reactivity while the geometry remains the same as that of Au_20_^51^. The 20-electron supershell closure in Au_20_ can be reproduced in a stable icosahedral *E*@${{\rm{Au}}}_{12}^{q}$ system, where *E* is a *p*-block element, through bonding-antibonding interactions between structural shells^52^. The adsorption energies of CO and O_2_ on Au_19_H are similar to those on the Au_20_ cluster^53^. More recently, selective doping with Hf and Ge atoms has been applied to modify the catalytic activity of Au_20_ through transforming the cluster structure^54^.

Although there have been some progress on the structural features and catalytic behavior of doped 20-atom gold clusters, the role of impurities in tailoring the magnetic properties of Au_20_ has been far less understood. Considering the fact that Cr, Mn, and Fe are interesting magnetic elements due to their unpaired 4*s* ^1^3*d* ^5^, 4*s* ^2^3*d* ^5^, and 4*s* ^2^3*d* ^6^ valence electrons, respectively, the interaction between these impurities and host electrons is expected to trigger essential changes in the magnetism of 20-atom gold clusters. In this study, we report results on searching for lowest-lying structures of gold clusters doped with a transition metal impurity Au_19_M (M=Cr, Mn, and Fe) and discussing the origin of their magnetic and stability properties. The finding results unravel the possibility of producing new magnetic superatoms in the golden pyramid’s family.

## Computational setup

The structural optimization of Au_19_M clusters have been carried out by density functional theory (DFT) calculations implemented in the Gaussian 09 software^55,56^. We used the BP86 functional in conjunction with basis sets cc-pvDZ-pp for Au and cc-pvDZ for Cr, Mn, and Fe. Possible structures of Au_19_M clusters were generated using a stochastic algorithm^57^. In addition, the local minima of previously-reported doped Au_*n*_ clusters also served as reference input. All guessing structures were initially optimized using the BP86 functional in jointing with cc-pvDZ-pp for Au atoms and cc-pvDZ for Cr, Mn and Fe dopants. Isomers having relative energies within 2.0 eV were selected for recalculating single point energies at the same functional but combining with larger basis set, cc-pvTZ-pp for Au and cc-pvTZ for Cr, Mn, and Fe. In the following, we discuss the structural, stability, and magnetic aspects of the doped Au_19_M clusters.

The selection of the BP86/cc-pvDZ-pp and cc-pvDZ functional resulted from test calculations for Au_2_, AuCr, AuMn, AuFe, Au_2_Cr^+^, and Au_2_Mn. The calculated results are presented in Table 1, along with available computational and experimental data for comparison. It can be seen that the used level provide reliable results for all of the examined properties. For example, the calculated spin states of all dimers and molecules are in excellent agreement with the other theoretical and experimental values^58–63^. The computed *D*_*e*_ of Au_2_, AuCr, and AuFe are 2.27, 2.27, and 2.20 eV, which are well consistent with the experimental ones, 2.29 ± 0.30, 2.28 ± 0.30, and 2.29 ± 0.30 eV, respectively. Although the predicted *D*_*e*_ of AuMn obtained in this work likely overestimates the experimental one, it is apparently in closer agreement with experiment than that of other calculations. It is worth to mention that the BP86 functional has been proved to yield the most reliable results for yttrium and vanadium doped gold species^64–66^. Therefore we are confident that the used computational approach is suitable to describe the structures and properties of Au_19_M clusters.

**Table 1:** Theoretical and experimental results of bond length *R*_*e*_ (Å), dissociation energy *D*_*e*_ (eV), ionization energy *IE* (eV), electron affinity *EA* (eV), and spin state *M* for Au_2_, AuCr, AuMn, AuFe, Au_2_Cr^+^, and Au_2_Mn.

System	Property	This work	Other calculations^a^	Experiments^b^
Au_2_	*R*_*e*_	2.52	2.56	2.47
	*D*_*e*_	2.27	2.14	2.29 ± 0.30
	*IE*	9.53	—	9.20 ± 0.21
	*AE*	2.08	1.82	1.92
AuCr	*D*_*e*_	2.27	—	2.28 ± 0.30
	*M*	6		
AuMn	*D*_*e*_	2.47	2.58	2.01 ± 0.22
	*M*	7	7	
AuFe	*D*_*e*_	2.20	—	2.29 ± 0.30
Au_2_Cr^+^	*M*	6	—	6
Au_2_Mn	*M*	6	6	—

^a^Refs^57–59.^

^b^Refs^60–62^.

## Results and Discussions

**Figure 1:**
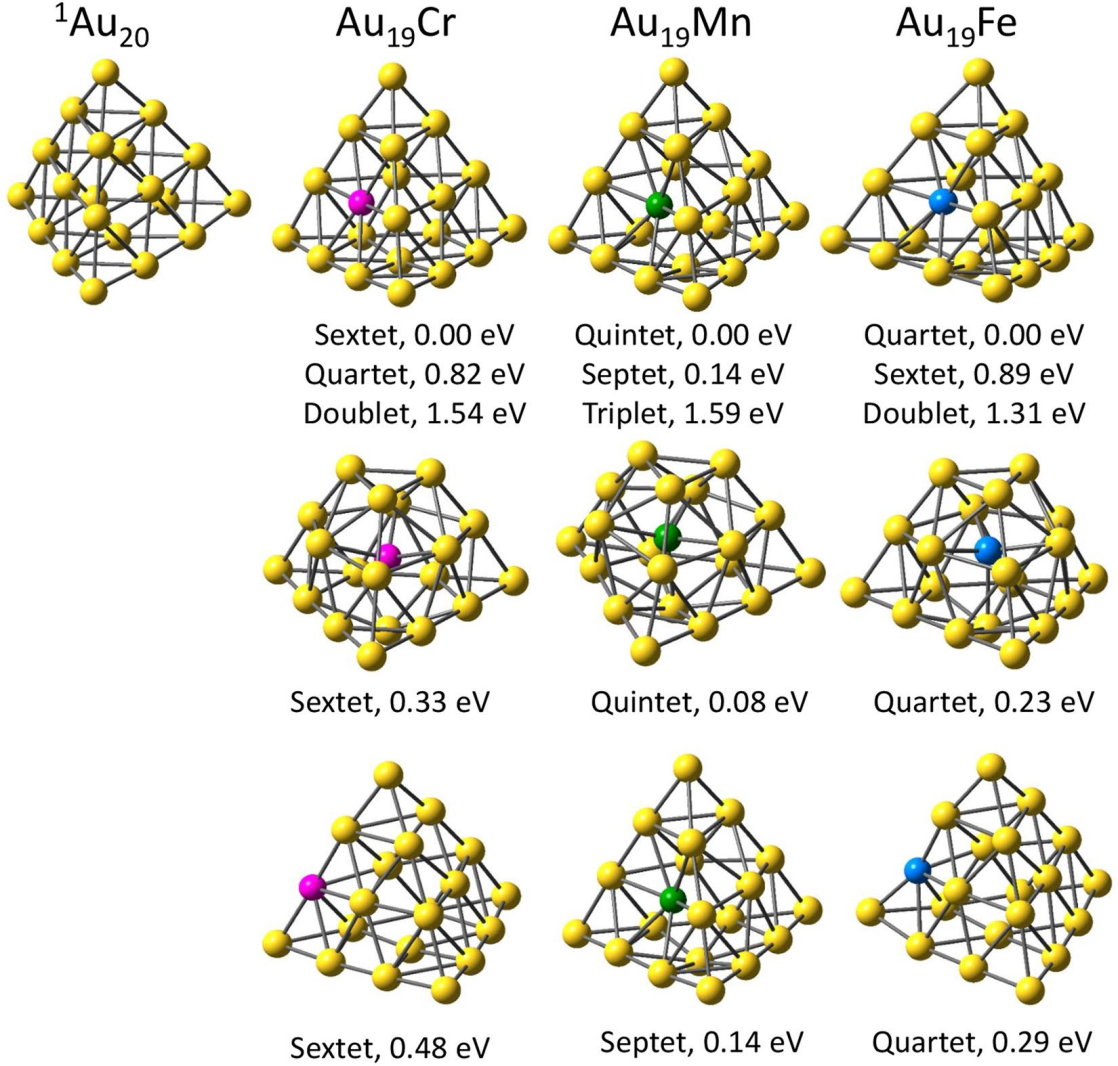
Optimized shapes and lowest-lying spin multiplicities of Au_19_M (M=Cr, Mn, and Fe) clusters with relative energies. The yellow, purple, green, and blue spheres represent Au, Cr, Mn, and Fe atoms, respectively.

The geometries of low-lying Au_19_M isomers with M=Au, Cr, Mn, and Fe are shown in Fig. 1. Although a large number of different structures and spin configurations are considered for each cluster, only the lowest-energy isomers are discussed. As expected, singlet Au_20_ is found to be very stable in form of a tetrahedral with a calculated HOMO-LUMO energy gap of 1.80 eV, in an excellent agreement with the measured value (1.77 eV)^12^. It is noteworthy that the ground-state geometry of all investigated clusters favor a slightly distorted tetrahedral form caused by the displacement of the impurity, where the dopant substitutes for a gold atom on the surface center. The second lower-lying isomers of Au_19_Cr, Au_19_Mn, and Au_19_Fe have the same motif of an endohedrally-doped truncated pyramid, lying at 0.33, 0.08, and 0.23 eV above their corresponding ground states, respectively. A tetrahedron with a substituted dopant atom on the edge serves as the next isomers of Au_19_Cr and Au_19_Fe with relative energies of 0.48 and 0.29 eV, respectively. On the other side, that of Au_19_Mn is a spin isomer (septet) of the corresponding ground state, locating at 0.14 eV higher in energy.

The most interesting feature of Au_19_M clusters is their magnetic behavior. Unlike the non-magnetic Au_20_, the ground state spin multiplicities of Au_19_Cr, Au_19_Mn, and Au_19_Fe clusters are sextet, quintet, and quartet, corresponding to total magnetic moments of 5, 4, and 3 *μ*_*B*_, respectively. The decreasing trend of the total magnetic moments with the increasing number of dopant’s valence electrons is noticeable. Other low-lying spin isomers are examined and listed in Fig. 1. It is found that the ground states of Cr and Fe doped Au_19_ clusters have relatively robust spin configurations. In particular, the next spin excitations of Au_19_Cr are quartet and doublet at 0.82 and 1.54 eV, respectively. For Au_19_Fe, at least 0.89 eV is needed to excite it from quartet to sextet states and 1.31 eV is required for the quartet-to-doublet transition. The spin stability of Au_19_Cr and Au_19_Fe is again reflected by the fact that their second and third lowest-energy isomers also favor the same spin states as the ground state ones. Whereas, the spin ground state of Au_19_Mn is less stable since an energy amount of 0.14 eV is sufficient to trigger a spin excitation.

**Figure 2:**
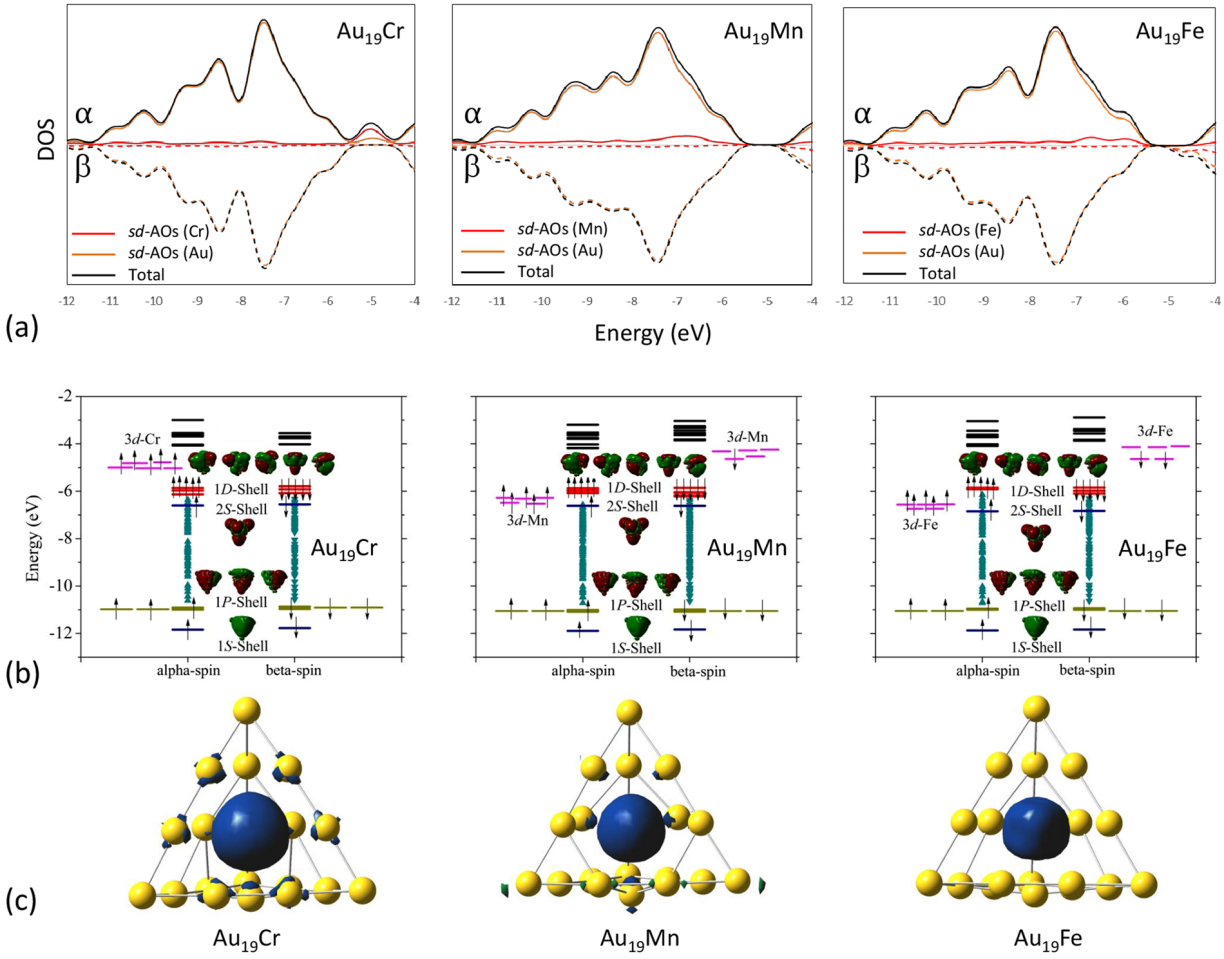
(**a**) Partial and total density of electronic states (DOS) and (**b**) one-electron-energy-level molecular diagrams (MO) of Au_19_M clusters. The black lines represent energy levels of the lowest unoccupied molecular orbitals (LUMO). The red, blue, dark yellow, and navy lines depict orbitals corresponding to the 1D, 2S, 1P, and 1S supershells, respectively. The magenta lines describe the localized 3*d*-M orbitals. The light blue triangles correspond for filled *d*-orbitals of Au atoms, which do not contribute to the supershell. Each upward (or downward) arrow represents one alpha-spin (or beta-spin) electron. (**c**) Total spin distribution of Au_19_M clusters plotted at a density of 0.004. The navy basins are for alpha-spin while the green ones are for beta-spin.

To gain insight into the magnetic behavior of Au_19_M clusters, the total/partial density of states (DOS), the molecular orbitals (MO) diagram, and the spin distribution of Au_19_M clusters are calculated and plotted in Fig. 2. Typically, when a metallic cluster is doped with a magnetic atom, the outermost orbitals of the magnetic impurity can hybridize with delocalized orbitals of the host to form supershells. Cr, Mn, and Fe atoms have electronic configurations of [Ar]4*s* ^1^3*d* ^5^, [Ar]4*s* ^2^3*d* ^5^, and [Ar]4*s* ^2^3*d* ^6^ corresponding to 6, 7, and 8 valence electrons, respectively. Assuming the each gold atom delocalizes one 6*s* electron, adding a Cr atom to the cluster of nineteenth gold atoms can create a pool of 25 itinerant electrons. Ordinarily, this forms an open shell of 2*P* ^5^ and does not lead to the observed spin state. However, there is another possibility in which valence orbitals of the Cr atom and delocalized MOs of the host hybridize and form a localized and a delocalized MO sets^67^. Once the delocalized MO set occupies states of itinerant electrons, the localized MO set has enough exchange splitting to cause a magnetic moment. With this picture in mind, the first 20 delocalized electrons of a Au_19_Cr cluster might be used to fill the molecular shell of 1*S* ^2^1*P* ^6^2*S* ^2^1*D* ^10^, which leads to the stable species. Its five remaining localized electrons are taken to partially fill the *d*-Cr atomic shell, which results in a total magnetic moment of 5 *μ*_*B*_. Similar argument can be applied for Au_19_Mn and Au_19_Fe, in which their delocalized electrons fulfill a shell configuration of 1*S* ^2^1*P* ^6^2*S* ^2^1*D* ^10^ and magnetic moments oscillates between 4 and 3 *μ*_*B*_, depending on the number of valence electrons in dopant atoms.

The results presented in Fig. 2 confirm the latter picture. The total/partial DOS [Fig. 2(a)] in combination with the MO diagram [Fig. 2(b)] indicate that the *s* orbitals from Au atoms play an essential role to the cluster shell orbitals while the half-filled *sd* orbitals of transition metal dopants become a key factor for the magnetic moment of studied species. In particular, the hybridized *sd*-M and *s*-Au electrons in Au_19_M clusters form both localized and delocalized electronic states. Similar to Au_20_, the delocalized states can be expressed in the full occupied electron shell sequence of 1*S* ^2^1*P* ^6^2*S* ^2^1*D* ^10^. Two low-energy DOS peaks at −11.9 and −11.0 eV response for the molecular shell orbitals 1*S* and 1*P* (navy and dark yellow levels), respectively. The molecular shell orbitals 2*S* and 1*D* (blue and red levels) that overlap with atomic orbitals *d* of Au atoms (light blue levels) are observed at −6.5 and 6.0 eV, respectively. Meanwhile, the localized states (magenta levels) are used to fill the atomic five *d* orbitals from the dopant atom with five, six, and seven electrons from Au_19_Cr, Au_19_Mn, and Au_19_Fe clusters, respectively. Since the delocalized states account for the 20-electron supershell closure, the unpaired electrons in the localized *d* orbitals cause the magnetic moments of clusters. It can be approximately considered that the chromium atom provides one electron to the cluster supershells and five others to the majority spin channel of *d* atomic subshell orbitals. Similarly, the manganese (iron) atom donates one electron to the cluster supershell orbitals, five electrons to the majority spin channel, and one (two) other(s) to the minority spin channel, respectively. According to the energy levels in MO diagrams, the electronic configurations of Au_19_Cr, Au_19_Mn, and Au_19_Fe clusters can be written as 1*S* ^2^1*P* ^6^2*S* ^2^3${d}_{\uparrow }^{5}$1*D* ^10^, 1*S* ^2^1*P* ^6^2*S* ^2^3${d}_{\uparrow }^{5}$1*D* ^10^3${d}_{\downarrow }^{1}$, and 1*S* ^2^1*P* ^6^2*S* ^2^3${d}_{\uparrow }^{5}$1*D* ^10^3${d}_{\downarrow }^{2}$, respectively. This picture can be further examined by mapping the spin distribution of studied Au_19_M clusters. As shown in Fig. 2(c), the total spin of the clusters is largely localized at the dopant atom, provided by the 3*d* state electrons as described in their aforementioned electronic configurations. A small amount of ferrimagnetic spin alignment adding to the total magnetic moments is found in Au atoms. Calculated local magnetic moments of Au_19_M clusters (not shown here) supports our argument that the total magnetic moments in Au_19_M clusters essentially result from the localized 3*d*-M orbitals.

Inheriting the major 20-electron supershell closing 1*S* ^2^1*P* ^6^2*S* ^2^1*D* ^10^ and highly symmetric geometry from the tetrahedral Au_20_, Au_19_Cr, Au_19_Mn, and Au_19_Fe are potentially stable species. Their dopant-dependent stability can be reflected through the comparison between corresponding binding energies per atom *E*_*b*_ with that of Au_20_. The calculated average binding energy of Au_20_ is found as 2.25 eV, which is in good agreement with the previously reported values^58^. Whereas, *E*_*b*_ of alloy species are slightly higher, that are 2.29, 2.30, and 2.31 eV for Au_19_Cr, Au_19_Mn, and Au_19_Fe clusters, respectively. Since the Au_19_M and Au_20_ systems have nearly identical geometries and similar *E*_*b*_ values, it could be suggested that the incorporation of transition metal dopants tends to stabilize the gold host and the stability of Au_19_M system can be relatively comparable to that of the robust Au_20_. We further examine the bonding nature between the dopant and Au host by analyzing the electron localizability indicator (ELI-D)^68^. The ELI-D isosurface of Au_19_M clusters produced at the bifurcation value of 1.0 are presented in Fig. S1 in the Supplementary Information file (Supplementary Information: The electron localizability of ground-state Au_19_M clusters). No localization domain is observed in the region between the dopant and Au_19_ host, implying that the dopant likely connects with Au moiety via ionic and/or highly polarized covalent bonds. A similar picture was also reported for Re@Au_11_Pt and Ta@Au_11_Hg superatomic systems, in which the energy decomposition analysis (ETS-NOCV) is used to emphasizes the importance of electrostatic and covalent interaction between metal dopant and host in stabilizing clusters^69^. To measure the chemical stability of the clusters, we examine the energy gaps between the LUMO of the minority (beta) spin and the HOMO of the majority (alpha) spin $[{\delta }_{1}=-({\varepsilon }_{HOMO}^{alpha}-{\varepsilon }_{LUMO}^{beta})]$ and between the LUMO of the majority (alpha) spin and the HOMO of the minority (beta) spin $[{\delta }_{2}=-({\varepsilon }_{HOMO}^{beta}-{\varepsilon }_{LUMO}^{alpha})]$. These values suggest the required amount of energy for an electron to jump from the HOMO of majority (minority) spin channel to the LUMO of minority (majority) one. The larger gap means the less reactive and more stable clusters^70–72^. Table 2 lists the HOMO and LUMO energies for the majority and minority spin channels and the values of *δ*_1_ and *δ*_2_ calculated for the ground states. Both energy gaps *δ*_1_ and *δ*_2_ take positive values, again confirming the inertness of all studied clusters. Notably, Au_19_Cr has the largest gaps of *δ*_1_ and *δ*_2_, which means that it is the most stable and least reactive species compared to the others and can be assigned as a magnetic superatom. Among clusters with higher doping concentration, Au_16_M_4_ would be a promising system due to its potential as a giant magnetic and highly symmetric superatom. Although the dopant atom apparently prefers substituting one Au atom in the surface center of singly doped species, the situation for Au_16_M_4_ clusters could be more puzzling. It is worth to mention that the unique stability of the golden pyramid Au_20_ can be understood in term of a superatomic molecule Au_16_Au_4_, where its superatomic core Au_16_ binds with four vertex Au atoms^42^. This approach has been supported by the recent findings of the golden pyramid’s smaller sisters, the tetrahedral ${{\rm{Au}}}_{17}^{+}$ and ${{\rm{Au}}}_{10}^{2+}$. These two systems were found highly stable in form of Au_13_ and Au_6_ octahedral cores, respectively, and the other four gold atoms are above four their triangular faces^37,73,74^. In this scenario, Au_16_M_4_ and its smaller magnetic counterparts, ${{\rm{Au}}}_{13}{{\rm{M}}}_{4}^{+}$ and ${{\rm{Au}}}_{6}{{\rm{M}}}_{4}^{2+}$, might be considered as a core in −4 charge state and four vertex M atoms. A careful examination of this interesting issue is perhaps warranted for future research.

**Table 2:** Calculated HOMO and LUMO energies (in eV) of the majority and minority spin channels and the values (in eV) of *δ*_1_ and *δ*_2_ for Au_19_M (M=Cr, Mn, and Fe).

Clusters	${{\boldsymbol{\varepsilon }}}_{{\boldsymbol{HOMO}}}^{{\boldsymbol{alpha}}}$	${{\boldsymbol{\varepsilon }}}_{{\boldsymbol{LUMO}}}^{{\boldsymbol{alpha}}}$	${{\boldsymbol{\varepsilon }}}_{{\boldsymbol{HOMO}}}^{{\boldsymbol{beta}}}$	${{\boldsymbol{\varepsilon }}}_{{\boldsymbol{LUMO}}}^{{\boldsymbol{beta}}}$	*δ*_1_	*δ*_2_
Au_19_Cr	−4.83	−4.06	−5.79	−4.05	0.78	1.73
Au_19_Mn	−5.86	−4.18	−4.44	−4.31	1.55	0.26
Au_19_Fe	−5.84	−4.08	−4.66	−4.10	1.74	0.58

## Conclusions

In summary, we have investigated structure, stability, and magnetic properties of singly doped Au_19_M (M=Cr, Mn, and Fe) clusters using DFT calculations. The most interesting point of our results is that all studied Au_19_M clusters favorably form magnetic copies of the golden pyramid Au_20_. The dopant atom is found to substitute for a gold atom on the surface center. The coexistence of both delocalized and localized electronic states in these clusters allows to fill the supershell orbitals and to simultaneously promote local magnetic moments, leading to stable magnetic systems. Au_19_Cr is the most stable species that can be assigned as a magnetic sister of the golden pyramid Au_20_.

## Electronic supplementary material

Supplementary Information
