# Supplementary material for: Au19M (M=Cr, Mn, and Fe) as magnetic copies of the golden pyramid
Source: Sci Rep. 2017 Nov 22;7:16086. doi: 10.1038/s41598-017-16412-3 (PMC5700080; doi:10.1038/s41598-017-16412-3)
Supplement: Supplementary file 1 — Supplementary Information [file 41598_2017_16412_MOESM1_ESM.pdf]

Au$_{19}$M (M=Cr, Mn, and Fe) as magnetic copies of the golden pyramid

Nguyen Minh Tam,[1,2,*] Ngo Tuan Cuong,[3] Hung Tan Pham,[4] and Nguyen Thanh Tung[5,]**

[1]Computational Chemistry Research Group, Ton Duc Thang University, Hochiminh, Vietnam
[2]Faculty of Applied Sciences, Ton Duc Thang University, Hochiminh, Vietnam
[3]Center for Computational Science, Hanoi University of Education and Training, Hanoi, Vietnam
[1]Department of Chemistry, KU Leuven, Celestijnenlaan 200F, B-3001 Leuven, Beligum
[5]Institute of Materials Science and Graduate University of Science and Technology, Vietnam Academy of Science and Technology, Hanoi, Vietnam
[*]Co-corresponding author: nguyenminhtam@tdt.edu.vn
[**]Corresponding author: tungnt@ims.vast.ac.vn

Supplementary Information

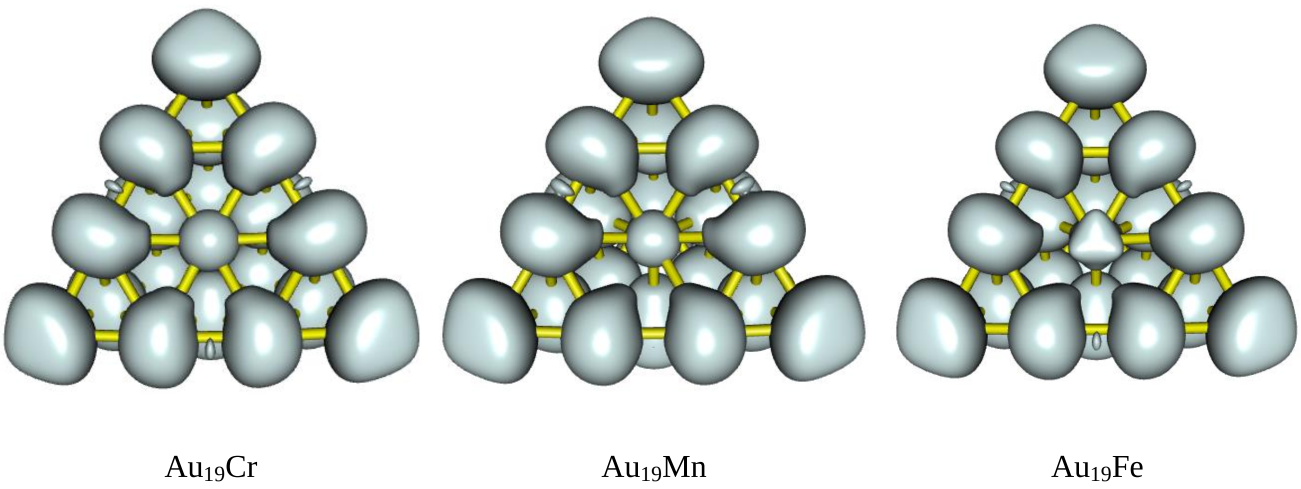

Figure S1. The ELI-D isosurfaces of ground-state Au$_{19}$Cr, Au$_{19}$Mn, and Au$_{19}$Fe clusters produced at the bifurcation value of 1.0.
